# Macrophage-Targeted Therapy: CD64-Based Immunotoxins for Treatment of Chronic Inflammatory Diseases

**DOI:** 10.3390/toxins4090676

**Published:** 2012-09-14

**Authors:** Dmitrij Hristodorov, Radoslav Mladenov, Michael Huhn, Stefan Barth, Theo Thepen

**Affiliations:** 1 Department of Experimental Medicine and Immunotherapy, Institute of Applied Medical Engineering, University Hospital RWTH Aachen, Aachen 52074, Germany; Email: dmitrij.hristodorov@rwth-aachen.de (D.H.); radoslav.mladenov@rwth-aachen.de (R.M.); michael.huhn@molbiotech.rwth-aachen.de (M.H.); barth@hia.rwth-aachen.de (S.B.); 2 Department of Pharmaceutical Product Development, Fraunhofer Institute for Molecular Biology and Applied Ecology, Aachen 52074, Germany

**Keywords:** CD64, macrophages, polarization, immunotoxins, inflammation

## Abstract

Diseases caused by chronic inflammation (e.g., arthritis, multiple sclerosis and diabetic ulcers) are multicausal, thus making treatment difficult and inefficient. Due to the age-associated nature of most of these disorders and the demographic transition towards an overall older population, efficient therapeutic intervention strategies will need to be developed in the near future. Over the past decades, elimination of activated macrophages using CD64-targeting immunotoxins has proven to be a promising way of resolving inflammation in animal models. More recent data have shown that the M1-polarized population of activated macrophages in particular is critically involved in the chronic phase. We recapitulate the latest progress in the development of IT. These have advanced from full-length antibodies, chemically coupled to bacterial toxins, into single chain variants of antibodies, genetically fused with fully human enzymes. These improvements have increased the range of possible target diseases, which now include chronic inflammatory diseases. At present there are no therapeutic strategies focusing on macrophages to treat chronic disorders. In this review, we focus on the role of different polarized macrophages and the potential of CD64-based IT to intervene in the process of chronic inflammation.

## 1. Introduction

**Figure 1:**
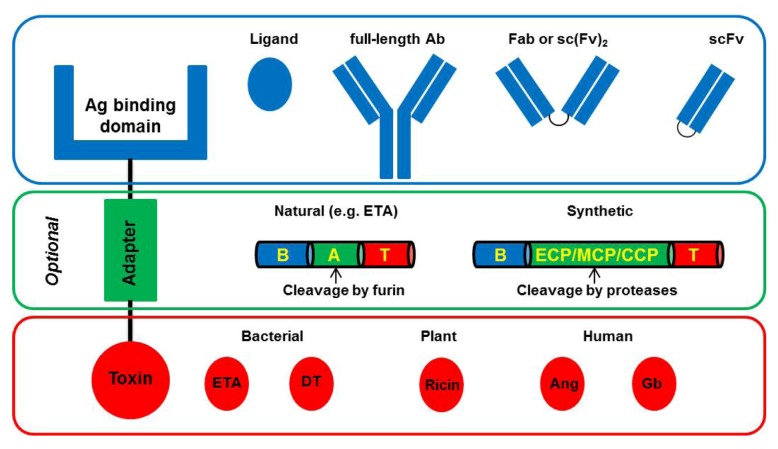
Basic architecture of Immunotoxins (IT).

Immunotoxins (IT) are chimeric proteins consisting of a binding domain, which is commonly an antibody or a derivative thereof, and a toxic domain, which is an enzyme usually derived from bacteria or plants (Figure 1). Abbreviations: Ag, antigen; Ab, antibody; Fab, fragment antigen binding; scFv: single chain fragment variable.

Classically, IT are comprised of a binding domain and a toxic domain, which optionally can be separated by an adapter peptide. The binding domain can be either a ligand (e.g., growth factor) or an antibody or a derivative thereof (e.g., scFv, Fab). The optional adapter peptide is used to improve translocation efficiency from the endosome into the cytosol. Naturally occurring adapter sequences can be derived from Pseudomonas Exotoxin A (ETA), which is composed of a binding domain (B), a translocation domain (A), and a toxic domain (T). Also, synthetic adapters containing an endosomal cleavable peptide (ECP), a membrane transfer peptide (MTP), and a cytosolic cleavable peptide (CCP) can be engineered. For cytotoxicity, bacteria-derived toxins (e.g., ETA, Diphtheria Toxin (DT)), plant-derived toxins (e.g., Ricin), or human enzymes (e.g., Granzyme B (Gb), Angiogenin (Ang)) can be used. 

After binding of the antibody to a target cell antigen, the IT is internalized followed by endosomal processing and final release of the toxin into the cytosol, where it induces cell death. In addition to antibodies as targeting units, several ligands including interleukins or growth factors have been used [1,2,3,4,5,6,7]. The internalization is almost exclusively dependent on the binding moiety and can vary strongly. In contrast, translocation to the cytosol can depend on several factors including the nature of the toxic domain, the design of the fusion protein (e.g., translocation-supporting peptide sequences between the binding and the toxic domain), and the presence of a *C*-terminal endoplasmic reticulum (ER)-retention signal [8,9,10]. For plant toxins, for example, the intracellular transport was shown to be regulated by sorting receptors cycling between the ER and the Golgi [8]. The cytotoxicity of both plant and bacterial toxins was also shown to be optimal when the toxic domain only translocates to the cytosol [11]. Separation of both domains is usually accomplished by proteases, such as furin, which are predominantly localized in the transreticular Golgi [12]. An alternative way of translocation is presented by the bacterial diphtheria toxin (DT). Upon internalization and pH-mediated unfolding of the protein, DT forms a hairpin, which inserts into the membrane of early endosomes and translocates to the cytosol [13,14]. All of these modalities need to be considered for optimal design of IT. 

In the beginning of IT development, antibodies were conjugated to the protein toxin via chemical linking, which however has many disadvantages like separate production and purification of targeting and toxic unit, low yields after conjugation, and a non-directed coupling leading to a heterogeneous protein preparation [15]. To overcome these and to allow the commercial development of IT, recombinant IT were generated by genetically fusing the ligand and the toxin resulting in a single chain DNA construct. To date, most IT can be simply expressed by fermentation of transformed *Escherichia coli* or by transiently transfected HEK293T cells and purified by standard chromatographic methods. One of the criteria for the toxic domain of the IT of choice is the ability to induce apoptosis. Induction of apoptosis as opposed to necrosis or pyroptosis is the preferred result of the IT, as it reflects a way of strongly regulated cell death without severely affecting the local environment [16,17]. The most prominent toxins which have been used so far are plant-derived ricin, especially the A chain thereof, bacterial Pseudomonas Exotoxin A (ETA), and DT [18]. Ricin belongs to the class II ribosome-inactivating proteins, which contain both binding and toxic domains and are therefore called holotoxins. Further members of this group are abrin, mistletoe lectin, and modeccin [19]. Plant toxins containing only a catalytic domain (e.g., saporin, bouganin, and gelonin) belong to the class I ribosome-inactivating proteins and are called hemitoxins [20]. All of these plant toxins have in common that they prevent the association of elongation factor (EF) 1 and 2 with the 60S ribosomal subunit [21,22]. 

In contrast, bacterial toxins, including ETA and DT, inhibit protein synthesis by enzymatically catalyzing the adenosine diphosphate ribosylation of EF2 in the cytosol [23,24]. Both bacterial toxins are multidomain proteins comprising a cell-binding and a toxic domain separated by a translocation domain. For use in IT, truncated versions of ETA and DT are generated by deletion of the cell binding domain [25,26,27,28,29]. This reduces the size of ETA and DT, respectively, making them even more suitable as fusion proteins and it increases their specificity preventing unwanted binding to healthy cells. The most prominent shortened version of ETA is PE38 (here referred to as ETA’) [27]. Historically, due to their relatively strong side effects, IT have been implicated for use in life threatening disease only and were therefore restricted to indications such as cancer. Recently, Madhumthi and Verma reviewed existing therapeutic targets for immunotherapy emphasizing that cancer, including solid tumors, lymphoma, and leukemia, represents the dominating indication for classical IT [30]. Besides their toxic side effects, immunogenicity of chimeric IT composed of a murine or human antibody and a bacterial or plant toxin had to be considered as an obstacle for treatment [31]. Generation of neutralizing antibodies by the immune system would reduce the number of possible treatment cycles. Different attempts have been done to reduce immunogenicity. For example, potential T- and B-cell epitopes on ETA have been identified and mutated expecting a less immunogenic version of the bacterial toxin [32,33,34]. An alternative strategy is to modify the IT using polyethylene glycol, which has been proved to be efficient in preventing immunogenicity of interferon and L-asparaginase [35,36,37]. However, these strategies up to now failed to significantly reduce immunogenicity. Vascular leak syndrome triggered by binding of toxins to endothelial cells represents another disadvantage of chimeric IT. As counter-measures, receptor mutation, inhibitors preventing the binding to endothelial cells and administration of anti-inflammatory agents have been taken [38]. Low or non-killing concentrations of such IT have also been reported to induce an enhanced inflammatory response via activation of innate immune sensors [39,40,41]. This fact is especially relevant in the context of treating inflammatory diseases as it would oppose the desired effect of resolving inflammation. A far more promising approach is presented by the generation of fully human cytolytic fusion proteins (hCFP). Fully human antibodies fused to human proteins, which are capable of directly or indirectly inducing apoptosis, are now gaining attention due to their safety. A dozen successfully applied hCFP already exist. Human RNases like RNase 1, 2, 3 and 5 (angiogenin), which degrade RNA and induce apoptosis by inhibition of protein synthesis, have been used to replace the non-human toxins [42]. Huhn *et al.* were able to show specific cytotoxicity of the human angiogenin to CD30 overexpressing Hodgkin lymphoma-derived cell lines delivered by a CD30 ligand (CD30L) [43]. Another hCFP CD30L-based IT was generated by Tur *et al.* who showed specific cytotoxicity of the human death associated protein kinase 2 towards several Hodgkin lymphoma cells *in vitro *[44,45]. Proapoptotic proteins such as Bik, Bak, Bax, DNA fragmentation factor 40, FAS-ligand, and TNF-related apoptosis-inducing ligand proofed effective in melanoma, renal cancer, cutaneous T cell lymphoma, and AML [46]. A novel IT composed of a scFv against CTLA4 and perforin was shown to kill CTLA-positive cell lines *in vitro *[47]. Although most of the IT were tested for toxicity on cancer cells, hCFP presenting a safe and non-immunogenic profile would allow for their application in diseases other than cancer. One interesting and, at the same time, challenging class of diseases which often lack effective and specific therapy are chronic inflammatory disorders. As macrophages are strongly involved in inflammation, they have been suggested to represent a suitable target for therapy.

## 2. The Role of Macrophages in Immunity

Macrophages belong to the mononuclear phagocytic system and are present in virtually all tissues. They originate from hematopoetic stem cells in bone marrow, which develop into blood circulating monocytes followed by a regulated migration into different tissues, either in steady state or upon inflammation. There, they finally differentiate into tissue-specific macrophages [48]. The nomenclature of terminally differentiated macrophages comprises among others osteoclasts in bone-marrow, microglial cells in the central nervous system, alveolar macrophages in lungs, Kupffer cells in the liver, and various forms of spleen macrophages [49]. These strongly phagocytic cells have long been considered to play a key role in the immune system. For the very first description of phagocytosis and its importance for immunity, Metchnikoff was awarded with the Nobel prize in 1908 [49,50]. This discovery has inspired immunologists to explore the function of macrophages as immune effector cells and to broaden the understanding of how these cells participate in direct host defense. Over time, their original function of pathogenic and homeostatic clearance, which includes the essential recycling of iron by engulfment of erythrocytes, and the removal of cell debris derived from tissue remodeling, apoptosis and necrosis, was extended to more complex physiological processes [49,51,52,53]. It is now known that they are potent inducers and regulators of immune responses and take a central role in orchestration of many metabolic functions in health and disease. Inflammation is one of the conditions, where macrophages play a critical role. The release of cytokines from apoptotic neutrophils, which are the first cells to infiltrate the site of inflammation [54], subsequently leads to the recruitment of macrophages which then guide the course of inflammation until resolution is achieved. In wound healing for instance, macrophages participate in all three major phases comprising inflammation, new tissue formation and tissue remodeling through communication with keratinocytes [55,56,57], fibroblasts and myofibroblasts [58,59,60,61,62], and endothelial cells [63,64,65,66]. For this very reason, macrophages play a key role in ensuring a physiologically normal inflammation. In consequence, pathology and proper resolution of inflammation are very close to each other in macrophage-coordinated events.

## 3. Classification of Polarized Macrophages and Their Contribution to Inflammation

**Figure 2:**
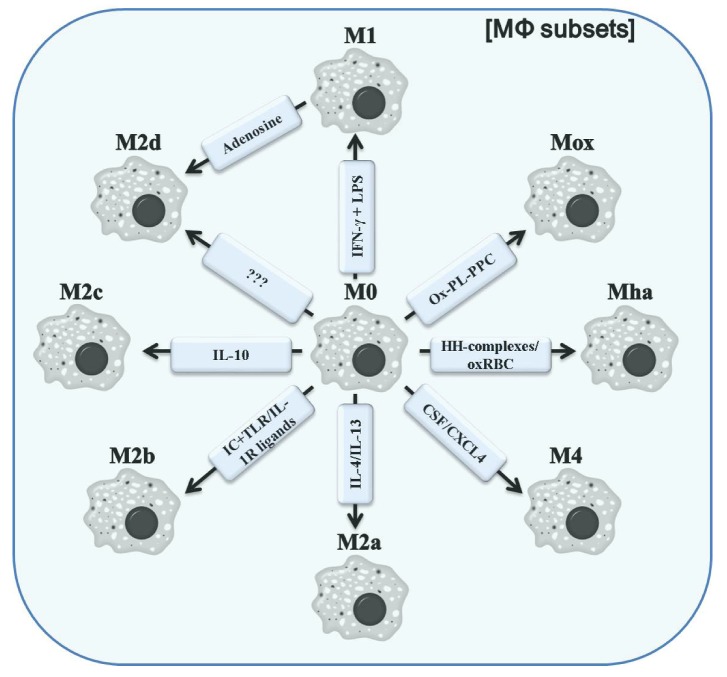
Different polarized MΦ subsets. Abbreviations: IC, immune complex; TLR, Toll-like receptor; IL-1R, IL-1 receptor; Ox-PL-PPC, ox-PL 1-palmitoyl-2arachidonoyl-*sn*-glycero-3-phosphorylcholine; HH-complexes, hapto-hemoglobin; oxRBC, oxidized red blood cells; CSF, colony stimulating factor; CXCL4, chemokine (C-X-C motif) ligand 4.

Inflammation is a complex and dynamic physiological process, which requires a well-balanced and controlled interaction of diverse cells. One of the cell types critically involved in initiation but also resolution of inflammation is macrophages. Across the different phases of an inflammatory response to e.g., infections or skin damage, their functions cover the production of pro-inflammatory cytokines and cytotoxic compounds, secretion of various growth factors to promote the function of other cells contributing to the resolution of inflammation, fighting invading microbes, and scavenging cell debris. In fact, these factors are neither produced simultaneously nor by the same kind of macrophages. Several recent reports have made clear that “the” macrophage does not exist, but that the term macrophage is rather used in a broader context for the description of a population of very versatile cells that share some common denominators. Due to their ability to adopt to a changing cytokine milieu and, vice versa, to influence the milieu by production of an array of soluble mediators, macrophages are nowadays considered to be very plastic and flexible cells [67]. Acquiring diverse functional phenotypes in response to environmental cues is reflected in the classification of polarized rather than activated macrophages. Mirroring the Th1/Th2 dichotomy, polarized macrophages were sub-divided into M1 and M2. M1 polarization, also referred to as classical activation, results from stimulation with Interferon-γ (IFN-γ), alone or in concert with bacterial lipopolysaccharide (LPS) [68]. The source of IFN-γ can be both innate and adaptive immune cells. Upon stress or first encounter to pathogens, natural killer (NK) cells, as part of the innate immunity, produce IFN-γ, thus polarizing macrophages to M1. In addition, an increased capacity of antigen presenting [69,70], these M1 cells are characterized by an enhanced microbicidal and tumoricidal activity mediated by the production of increased levels of superoxide anions, and oxygen and nitrogen radicals, which in summary confers direct host resistance to infections [71,72,73]. However, the capability of NK cells to provide sustainable amounts of IFN-γ is limited. Since M1 macrophages induce a Th1 response by secretion of high levels of pro-inflammatory cytokines, such as interleukin (IL)-12, IL-23, and tumor necrosis factor (TNF)-α, the polarization of macrophages to M1 can be amplified by a continued supply with IFN-γ by T cells. In contrast to T cells, which act in an antigen-specific manner, M1 polarized macrophages kill unspecifically, merely restricted by the distance to the target cell. While M1 macrophages represent the one extreme of polarization, the second extreme is summarized under the term alternatively activated macrophages or M2 macrophages [52]. During the past decade, this term has been expanded to a more heterogeneous cell population [74,75]. The nomenclature of alternatively activated macrophages is somewhat confusing and changes over time. Terms used are: M2, alternatively activated macrophages, type II activated macrophages, deactivated macrophages, M2a, M2b and M2c and a few more. In effort to find a more informative and clear classification of polarized macrophages Mosser and Edwards [49] suggested the following three sub-populations based on their functions: host defense, wound healing and immune regulation. This classification is in accordance with that proposed by Mantovani *et al.* [69], where M1 would refer to host defense, M2a to wound healing and M2b together with M2c to immune regulation. For discrimination of different macrophage phenotypes, Mantovani *et al.* not only considered the function, but also the respective stimuli generating the sub-populations. Thereby, M2a is produced by IL-4 or IL-13 both of which activate STAT6 via binding to the IL-4 α-subunit, which is shared in both receptors [76,77,78,79,80]. M2b results from a combined exposure to immune complexes with Toll-like receptor (TLR)- or IL-1 receptor (IL1R)-ligands, whereas M2c is induced by IL-10 [69]. Supplementary to Mossers and Edwards’s classification, Mantovani *et al.* discriminate between M2b macrophages, which in addition to immunoregulatory functions also induce Th2 driven inflammation, and M2c macrophages, which are thought to be predominantly responsible for negative/deactivating immunoregulation. More recently, additional sub-populations of macrophages were discovered (Figure 2).

Diverse ways of polarizing macrophages are induced by distinct signals from the microenvironment. Unpolarized macrophages/monocytes (M0) acquire a M1 phenotype upon stimulation with IFN-γ and LPS. M2a macrophages, especially responsible for wound healing, result from exposure to IL-4 or IL-13. Other members of the alternatively activated macrophages include M2b (after exposure to IC + TLR-ligands or IL-1R ligands) and M2c (polarized by IL-10). M2d represents a more recently discovered macrophage subset, which results from re-polarization from M1 by adenosine signaling. Whether an initial stimulus polarizing M0 to M2d does exist is yet unknown. The apparently related subsets Mox and Mha can be generated *in vitro* by stimulation with Ox-PL-PPC and HH-complexes or oxRBS, respectively. M2 macrophages are a hydride-type subset with combined features of M1 and M2 macrophages. These cells were shown to be CSF/CXCL4-dependent. 

**Figure 3:**
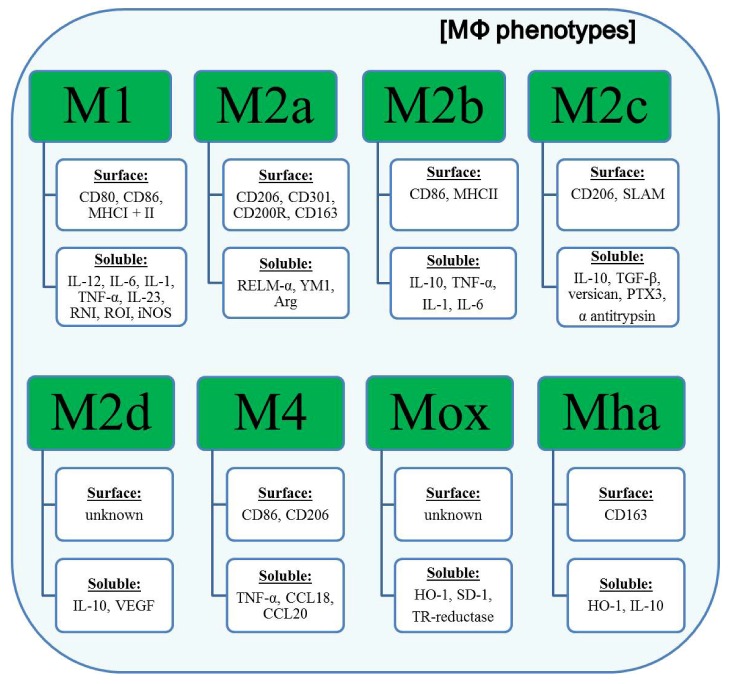
Phenotypes of different polarized MΦ.

In the context of atherosclerosis, a population of macrophages expressing a unique set of genes including Heme oxygenase-1 (HO-1), sufiredoxin-1, and thioredoxin-reductase in a nuclear factor, erythroid-derived 2, like 2(Nrf2) dependent manner was found within the aortas of 30 week western diet-fed *Ldlr*−*/*− mice by immunohistochemistry and flow cytometry. *In vitro*, this phenotype could be induced by ox-PL 1-palmitoyl- 2arachidonoyl-sn-glycero-3-phosphorylcholine resulting in the designation as Mox macrophages [81]. Furthermore, Mox macrophages seem to be closely related to the lately proposed hemorrhage-associated macrophages (Mha) [82,83]. Human monocytes can be differentiated to Mha macrophages *in vitro* using hapto-hemoglobin complexes or oxidized red blood cells leading to the up-regulation of CD163, HO-1 and IL-10 in an Nrf2-dependent manner. Moreover, a CSF/CXCL4-dependent macrophage subset, discovered in 2010 by Gleissner *et al.* [84], was termed M4. These macrophages were shown to be characterized by a mixed, but unique profile of transcripts including higher levels of CD86 and TNF-α (both M1-like), CD206, CCL18 and CCL20 (all M2-like) and lower levels of pentraxin 3 (PTX3), CD36 and IL-10 (M2-like). Although these cells have a weak phagocytic activity, the function of M4 macrophages remains poorly understood. Finally, the laboratory of Samuel Joseph Leibovich has characterized one further M2 subtype, termed M2d, which unlike previously described M2-like macrophages, are the result of a switching event from M1 macrophages as response to adenosine A_2A_ receptor signaling induced by TLR agonists [85]. M2d macrophages were demonstrated to markedly decrease the expression of pro-inflammatory cytokines including IL-12 and TNF-α, while concurrently producing high levels of IL-10 and vascular endothelial growth factor (VEGF). Interestingly, the classical markers of wound healing or M2a macrophages were not up-regulated pointing to an M2c-resembling phenotype of M2d macrophages. The phenotypes of different polarized macrophages are summarized in Figure 3.

Polarized macrophages show characteristic expression profiles of surface receptors and soluble mediators. M1 macrophages are characterized by an elevated expression of the co-stimulatory receptors for T cell activation CD80 and CD86 and both MHC molecules underlining their role as potent antigen presenting cells. On the soluble level, M1 macrophages up-regulate the expression and secretion of pro-inflammatory cytokines, such as IL-12, IL-6, and TNF-α. In contrast, M2 (a-d) macrophages preferentially produce anti-inflammatory cytokines (e.g., IL-10). Another hallmark of M2 macrophages (especially M2a and M2c) is the up-regulation of the mannose receptor (CD206). Recently identified macrophage subsets (M4, Mox and Mha) have until now been poorly characterized. Abbreviations: MHCI/II, major histocompatibility complex I/II; SLAM, signaling lymphocytic activation molecule; RNI, reactive nitrogen intermediates; ROI, reactive oxygen intermediates; iNOS, inducible nitric oxide synthase; RELM-α, resistin-like molecule α; YM1, heparin binding lectin; Arg, arginase; TGF-β, tumor growth factor β; PTX3, pentraxin 3; VEGF, vascular endothelial growth factor; CCL18/20, chemokine (C-C motif) ligand 18/20; HO-1, heme oxygenase-1 (HO-1); SD-1, sufiredoxin-1; TR-reductase, thioredoxin-reductase.

Together, this huge diversity in alternatively activated macrophages illustrates how different physiological processes might be accurately regulated by distinct macrophage subsets depending on the environmental conditions given. As already exemplified for M2d, all polarization subsets are also believed to be plastic and to undergo phenotypical and functional switches upon signals from the milieu, thus making the versatility of the macrophage system even vaster than that which emerges from the initial polarizing stimulus. However, imbalance and/or dysregulation of macrophage-controlled processes are strongly associated with a pathological outcome including several autoimmune diseases, impaired wound healing, metabolic disorders, and cancer [86,87,88,89,90,91,92,93]. Mediated by an autocrine secretion, certain macrophage subsets sustainably maintain the cytokine milieu leading to the persistence of these cells for an inappropriate period of time. In addition, T cells become activated and further promote the inflammatory status. Both cell types, T cells and macrophages, are initiators of different inflammatory diseases and can be used as targets in diagnosis and therapy. Based on the recently acquired knowledge on the involvement of distinct polarized macrophages in certain diseases and the anticipated rapid increase of novel findings in this field, polarized macrophages are more and more becoming an object of interest. Selective depletion of distinct macrophage populations, without adversely affecting others would allow for selective therapy with fewer side effects. However, polarization of macrophages represents a fairly new field of research and little is presently known on the origin, function, and plasticity of different polarized macrophages. So far, no effective therapies selectively targeting one population of macrophages do exist.

## 4. Improved CD64-Targeting Immunotoxins for Therapy of Chronic Inflammatory Diseases

Due to the demographic transition towards an overall older population, which is expected to continue growing in the next years, age-associated disorders are becoming more and more relevant to the welfare of our population. Following this tendency, the ratio of care-dependent people to care assistants is anticipated to increase significantly. As a result, the economic burden for the public health system will be devastating. Among cardiovascular diseases and cancer, disorders caused by chronic inflammation also belong to the class of age-associated widespread diseases. However, since no specific targets exist for most of the inflammatory diseases, current medical therapy relies on primarily facilitative and symptomatic rather curative treatments. Treatment of rheumatoid arthritis, for example, consists of blocking the effect of TNF-α, a pro-inflammatory cytokine secreted by activated inflammatory macrophage, using monoclonal antibodies, such as infliximab, etanercept or adalimumab [94]. Mesalamine or 5-aminosalicyl acid is another anti-inflammatory drug which is routinely used for treatment of inflammatory bowel disease, including ulcerative colitis and Crohn’s disease [95,96]. Targeting and depleting T cells via IL-2 receptor [97], regulated on activation normal T cells expressed and secreted (RANTES) [98], CCR5 [99], CD5, VLA-4, CD69, CD44, CD40 ligand and Ox-40 have been implicated in therapy of inflammatory diseases including psoriasis and rheumatoid arthritis, renal diseases, multiple sclerosis, diabetes, graft versus host disease, systemic lupus erythematosus, autoimmune dermatoses and oophoritis, to name just a few. 

However, clinical management consisting of blocking pro-inflammatory cytokines target the amplification loop of inflammation by depleting secondary inflammatory signals (e.g., TNF-α). Beyond that, depletion of specific subsets of antigen specific T cells is associated with the undesirable reduction of the immune systems diversity. An intervention in processes that do not amplify, but rather cause the imbalance of pro- and anti-inflammatory cytokines would potentially result in a more rational approach to suppress chronic inflammation. One suitable target for this approach are macrophages which substantially contribute to chronicity by production of pro-inflammatory soluble mediators. To allow specific targeting of these cells, a surface molecule had to be selected to act as an entrance mediator. Screening of surface expression revealed FcγRI, also referred to as CD64, to be up-regulated in macrophages. Compared to the other three Fcγ receptors (*i.e.*, FcγRII (CD32), FcγRIII (CD16) and neonatal FcγR), CD64 displays several exclusive properties: (1) high affinity; (2) ability to bind and internalize monomeric IgG [100]; and (3) constitutive expression only on macrophages, monocytes and their progenitors [101,102,103]. These properties make CD64 a suitable target molecule for a selective therapy directed against macrophages. First evidence for the success of this strategy was given by Thepen *et al.* in 2000, when elimination of CD64-positive macrophages using the IT H22-RicinA was demonstrated to resolve chronic skin inflammation in transgenic mice within 24 h [104]. In addition, Thepen *et al.* also showed by histological examination that other inflammatory cells disappeared from the site of inflammation and clinical parameters like vascular leakage and increased temperature were restored. Six years later, the same laboratory showed the inhibition of progression of arthritis in a rat model [105] and efficient killing of activated macrophages from synovial fluid obtained from human rheumatoid arthritis patients using the same IT [105]. The therapeutic approach of targeting macrophages by H22-based IT was then transferred to an ischemia-reperfusion rat model, where Fet *et al.* showed that treatment with H22(scFv)-ETA’ was effective to preserve renal function and morphology and ameliorated ischemia-induced kidney injury [106]. Although elimination of activated macrophages proofed effective in the treatment of inflammatory diseases, this strategy needs to be considered with caution when transferred to other macrophage-associated diseases. For example, during pulmonary tuberculosis in mice macrophages were shown to have a dual function. While non-specific depletion of all macrophages using liposomes improved clinical outcome, specific elimination of activated macrophages only led to impaired resistance to infection, reflected by enhanced mycobacterial outgrowth [107]. This study gives a further example for the existence of distinct macrophage populations, each fulfilling another function. 

The successful application of H22-RicinA and H22(scFv)-ETA’ in different therapeutic approaches, not only the inflammatory diseases described above, but also cancer, has moved forward the development of IT containing potentially immunogenic plant- or bacteria-derived toxins to a new generation of hCFP. The rational approach of selecting human enzymes suitable to act as a toxin was based on their ability to induce apoptosis in target cells. Stahnke *et al.* found human Granzyme B to confer specific killing to primary CD64-positive cells from an acute myeloid leukemia (AML) patient *ex vivo* and to AML-related cell line U937 *in vitro* after delivery by H22(scFv). The cytotoxicity was shown to be mediated by activation of caspase-3 leading to the induction of apoptosis and the half-maximal inhibitory concentration (IC_50_) was in the range of 1.7–17 nM, underlining the efficacy of the hCFP [108]. To further exploit possible strategies to improve the performance of hCFP, two critical parameters were investigated: (1) internalization and (2) translocation from endosomes into the cytosol. Although cross-linking is not required for internalization of CD64, it is known to be an enhancing factor [102,109]. Therefore, a bivalent construct was engineered resulting in H22(scFv)_2_-IT. Indeed, this IT proofed more effective *in vitro* and in a SLS induced chronic cutaneous inflammation model in transgenic mice *in vivo *[110]. The second and more sophisticated adaptation was the insertion of short translocation-supporting peptide sequences between the *C*-terminus of the scFv and the *N*-terminus of a human enzyme. Translocation is one of the bottlenecks when applying IT via the receptor-mediated endocytosis pathway and is therefore a crucial part of IT development. This fact is especially true for human enzymes as they originally do not contain a translocation domain, which is in contrast to bacterial toxins (e.g., ETA). In fact, Hetzel *et al.* demonstrated improved efficacy of H22(scFv)-Angiogenin by insertion of small cleavable adapter sequences containing a synthetic translocation domain flanked by proteolytically cleavable endosomal and cytosolic consensus sites. Consequently, enhanced translocation resulted in 20-fold cytotoxicity and a superior serum stability of the hCFP [111].

Taken together, targeting macrophages by IT has been repeatedly shown to be a suitable tactic for treatment of chronic inflammatory diseases and also cancer. The presence of different types of macrophages at different phases of chronic inflammation would make the targeting of the distinct sub-populations an attractive therapeutic approach. However, treating chronic disorders requires repetitive administration of the therapeutic, which in case of not fully human IT would lead to immunogenicity, thus making the treatment ineffective. In addition, approval of therapeutics containing bacteria- or plant-derived components would also present a challenging, if not an impossible task. To overcome these drawbacks, hCFP were developed over time and proofed effective for different indications *in vitro* and *in vivo*.

## 5. Conclusions

IT have emerged as a powerful tool for targeted therapy of many diseases. Due to unspecific cytotoxicity, immunogenicity and other undesired side effects, the use of IT in the clinic has been predominantly limited to treatment of life-threatening diseases, such as cancer, only. However, new generation IT, which are now composed of fully human proteins, overcome these disadvantages resulting in an increased application range of these hCFP. New indications also cover the class of chronic inflammatory disorders. Thereby, elimination of macrophages rather than antigen-specific T cells as major producers of inflammatory cytokines was demonstrated to be a more indulgent principle of how an impaired inflammation can be brought under control. Currently, polarization of macrophages, their phenotypes and functions, and most importantly, their involvement in diseases are being extensively studied. Due to their plasticity, macrophages undergo phenotypical and functional switches during the course of the disease. Identification of distinct macrophage subsets, which are critically involved in the pathology of the disease, and development of suitable targeting approaches for elimination of these macrophages would allow for an effective and side effect poor treatment of many inflammatory and autoimmune disorders. Moreover, the assignment of one specific macrophage sub-population is not limited only to inflammation. For instance, M2c macrophage are thought to be the so-called tumor-associated macrophages supporting the tumor cells in escaping from the immune system and promoting the vascularization of the tumor body. We anticipate that in near future, it will be possible to identify strategies to target individual subsets of macrophages without adversely affecting the pathology-unrelated populations of macrophages. 
